# Impact of recipient and donor factors on corneal graft clearance: insights from serial anterior segment optical coherence tomography

**DOI:** 10.1016/j.ajoint.2025.100147

**Published:** 2025-06-18

**Authors:** Stylianos Christodoulou, Dimitris Kola, Fedonas Herodotou, Aikaterini Athanasiadou, Chara Tzavara, Neofytos Michael, Anastasia Neokleous, Georgina Hadjilouka, Sotiria Palioura

**Affiliations:** a Ophthalmology Clinic, Archbishop Makarios III Hospital, State Health Services Organization, Nicosia, Cyprus; b Department of Ophthalmology, University of Cyprus Medical School, Nicosia, Cyprus; c Athens Eye Experts Ophthalmology Practice, Athens, Greece; d Department of Biostatistics, National and Kapodistrian University of Athens Medical School, Athens, Greece; e Department of Ophthalmology, Bascom Palmer Eye Institute, University of Miami Miller School of Medicine, Miami, Florida, USA

**Keywords:** AS-OCT, DSAEK, PKP, Central corneal thickness, Graft thickness

## Abstract

**Purpose:**

To evaluate how recipient and donor characteristics influence corneal graft clearance, using serial measurements of central corneal thickness (CCT) and central graft thickness (CGT) derived from anterior segment optical coherence tomography (AS-OCT).

**Design:**

Prospective cohort study.

**Methods:**

Seventy-one patients (76 eyes) who underwent corneal transplantation (October 2021–April 2024) were followed for at least six months. Serial AS-OCT scans were used to extract CCT and CGT measurements, which were analyzed using mixed linear regression models with time splines to assess changes over time. These thickness parameters served as surrogate markers of corneal and graft deturgescence, respectively.

**Results:**

Both CCT and CGT (for DSAEK grafts only) decreased significantly up to 3 months post-surgery (β = −1.73 μm/day, SE = 0.12, p < 0.001; β = −0.53 μm/day, SE = 0.05; p < 0.001), stabilizing thereafter (β = 0.063 μm/day, SE = 0.045, p = 0.156; β = 0.02 μm/day, SE = 0.02; p = 0.228). Faster clearance was observed in grafts from younger donor age (<56 years; β = 0.67 μm/day, SE = 0.23; p = 0.004) and those with higher endothelial cell density (ECD ≥3,021 cells/mm^2^; β = –1.14 μm/day, p = 0.001). Penetrating keratoplasty was associated with slower CCT reduction compared to Descemet stripping automated endothelial keratoplasty (β = 0.74 μm/day, SE = 0.32; p = 0.021). Other preoperative factors did not significantly influence clearance

**Conclusion:**

CCT and CGT are useful quantitative markers of corneal graft clearance. Donor age, ECD and surgical technique significantly influence early postoperative dynamics. Serial AS-OCT imaging offers valuable, non-invasive insights into graft behavior that can inform clinical decision-making.

## Table of contents statement

This study evaluates the impact of pre-operative recipient and donor-related factors on corneal graft clearance rates using serial anterior segment OCT imaging. Findings highlight the significance of donor age and endothelial cell density in early recovery, providing critical insights into optimizing graft success and post-operative management.

## Introduction

The success of corneal grafts is influenced by a combination of recipient-, surgical-, and donor-related factors, yet the rate at which grafts clear and stabilize remains variable and understudied. ^1–3^ While partial-thickness techniques such as Descemet stripping automated endothelial keratoplasty (DSAEK) and Descemet membrane endothelial keratoplasty (DMEK) are increasingly preferred for their favorable recovery profiles^4,5^, variability in graft clearance rates remains an important clinical challenge. Traditionally, research on graft success has focused on long-term outcomes such as endothelial cell density (ECD) or graft failure, while early post-operative dynamics—particularly graft deturgescence—have received less attention. Understanding the determinants of early graft deturgescence is essential for optimizing postoperative outcomes.

Anterior segment optical coherence tomography (AS-OCT) has emerged as a vital tool in this context. AS-OCT provides high-resolution pachymetric maps and cross-sectional images of the cornea, allowing for detailed and continuous assessment of graft anatomy and health over time. Its non-invasive nature and ability to produce repeatable and reproducible measurements make it an invaluable tool in both preoperative planning and post-operative follow-up of corneal grafts.^6,7^ This technology provides a valuable opportunity to assess early graft behavior and identify predictors of faster or slower edema resolution.

Both recipient pre-operative factors, such as the indication for surgery, as well as donor-related variables, such as donor age, endothelial cell density (ECD), and death-to-preservation time (DPT), have been shown to impact graft clarity and longevity in multiple studies over the years. The endpoint of such research efforts has traditionally been graft survival or ECD at a certain time point after transplantation.^3,8–11^

In this study, we are taking advantage of AS-OCT’s capability to provide detailed pachymetric imaging of the whole cornea and of the graft itself in order to quantify the rate of graft clearance and evaluate how donor and recipient characteristics influence early post-operative dynamics. Results are presented for all grafts in this study (i.e. PKP, DSAEK, DMEK), while a subgroup analysis for DSAEK grafts only is also performed. We hypothesize that younger donor age, higher ECD, and shorter DPT are associated with faster clearance of corneal edema, as measured by the rate of decrease in central corneal thickness (CCT) and central graft thickness (CGT).

## Methods

### Study design & participants

This prospective cohort study included patients who underwent corneal transplantation [DMEK, DSAEK, or penetrating keratoplasty (PKP)] by a single surgeon (SP) between October 2021 and April 2024. All patients had a minimum follow-up of 6 months, with serial AS-OCT scans performed at fixed postoperative intervals. Exclusion criteria included grafts with operative complications and/or need for rebubbling postoperatively, cases of rejection or failure during the study period as well as grafts with less than 6 months of follow-up. The study was approved by the Institutional Review Board and adhered to the tenets of the Declaration of Helsinki. Informed consent was obtained from all participants prior to enrolment.

### AS-OCT imaging protocol

Participants underwent AS-OCT imaging (Triton, Topcon Healthcare, Tokyo, Japan) pre-operatively and at fixed post-operative time intervals: day 0 (at four hours post-surgery), day 1, week 1, month 1, month 3, month 6, month 9 and every 6 months thereafter. The scans provided high-resolution pachymetric maps of the central 6 mm zone and cross-sectional images of the entire cornea and graft. The pachymetry maps of the central 6 mm zone were used to automatically calculate CCT. In contrast, CGT was manually measured on cross-sectional images using the caliper tool in the OCT software. CGT measurements were reviewed by two independent masked observers to ensure accuracy and consistency in measurements; in cases of discrepancy, a consensus measurement was recorded. Pre-operative donor graft thickness was measured by the eye bank immediately before tissue dissection and shipment.

### Outcome measurements

The primary outcomes were the rates of corneal and graft clearance, assessed by changes in CCT and CGT (for DSAEK grafts) over time, respectively. Pre-operative variables included recipient age, recipient pre-operative diagnosis, and recipient pre-operative CCT. Donor-related variables included donor age, donor graft ECD, and donor graft DPT. Additional variables such as time from death to surgery (donor graft death-to-surgery time), donor diabetes status, and cut-to-surgery time (for DSAEK grafts) were also recorded and correlated with the rate of corneal and graft clearance.

### Statistical analysis

Quantitative variables are summarized as mean (standard deviation) and median (interquartile range), while categorical variables are presented as absolute and relative frequencies. When patients contributed both eyes, a random effect for patient ID was included in the mixed linear models to account for within-subject correlation. The Kolmogorov-Smirnov test was used to assess the normality of quantitative variables. Variables showing skewed distributions or significant deviation from normality were summarized using medians and interquartile ranges.

To assess longitudinal changes in recipient post-operative CCT and post-operative CGT (for DSAEK grafts), mixed linear regression models with a spline for time (knot placed at 3 months) were fitted that account for multiple measurements per transplant, obtained at different time points and allowing for missing values. Adjusted regression coefficients (β) with standard errors (SE) were computed from the mixed model analyses. Interaction effects for the periods less than and greater than 3 months postoperatively were also evaluated, and coefficients only for significant interactions are reported.

In these models, a significant negative time coefficient represents a reduction in the dependent variable (CCT or CGT) over time. Interaction terms were included to assess whether this rate of change differed across donor or recipient characteristics. A negative interaction coefficient indicates a steeper decline (i.e., faster clearance), while a positive interaction term suggests a slower decline. For example, although both younger donor age and higher ECD were associated with faster clearance, the direction of their respective β coefficients reflects how each modifies the slope of thickness reduction over time. Main effects represent baseline group differences, while interaction terms capture differential temporal trends.

The 3-month knot point for the time spline was selected based on clinical rationale, reflecting the typical timeframe for graft deturgescence stabilization. This choice was further supported by visual inspection of the data, which showed a plateau in thickness changes beyond 3 months. Alternative knot placements (e.g., 2 and 4 months) were tested in sensitivity analyses and produced comparable model estimates without improving interpretability or fit. CCT and CGT were analyzed using separate mixed linear regression models. This approach was chosen because CGT is relevant only to DSAEK grafts, whereas CCT was evaluated across all surgical types. Separate modelling allowed us to account for differences in graft types and optimize interpretation for each outcome. Missing data were handled using mixed linear regression models, which accommodate incomplete longitudinal data under the assumption of missing at random (MAR). No imputation was performed; all available observations at each time point were included in the analyses.

Analyses for recipient post-operative CCT and CGT by donor age, donor graft ECD and donor eye bank-measured graft thickness were performed using both univariable and multivariable mixed linear models. Donor variables were dichotomized at their median values (56 years for age, 3,021 cells/mm^2^ for ECD, and 68.5 μm for graft thickness) to enhance interpretability. For both outcomes (CCT and CGT), adjustments for relevant covariates, including their preoperative values, donor graft ECD, recipient age, preoperative diagnosis, donor diabetes status, death-to-preservation time, death-to-surgery time, and cut-to-surgery time were made. Type of surgery was additionally included for CCT analysis. Model diagnostics were performed to ensure adequacy: residuals were visually assessed for normality using Q–Q plots and histograms, and for homoscedasticity using residual-versus-fitted plots. Multicollinearity was evaluated using variance inflation factors (VIFs), all of which remained below accepted thresholds. While CCT and CGT were used as quantitative surrogate markers of graft clearance, all grafts included in the study were clinically clear at each postoperative visit. However, formal correlation analyses between pachymetric measurements and clinical graft clarity or visual acuity were not performed.

All reported p values are two-tailed. Statistical significance was set at p<0.05 and analyses were conducted using the STATA statistical software (version 15.0).

## Results

A total of 76 corneal transplants were analyzed. The baseline characteristics of the sample are summarized in Table 1. Among recipients, 51.3 % were male, with a mean age of 73.8 years (SD = 12.0) and a median age of 74 years. Donors had a mean age of 54.0 years (SD = 15.9) and a median age of 56 years. Pseudophakic or aphakic corneal edema (PACE) was the most common pre-operative diagnosis, accounting for 41.7 % of cases, and the majority of the procedures (75 %) were DSAEK.

In terms of donor characteristics, 64.5 % were male and 35.5 % were female. A history of diabetes mellitus was present in 17.3 % of donors. Optisol-GS was used as the preservation media in 86.9 % of cases, and Eusol-C in the remaining 13.1 %. The preservation solution used was Optisol-GS In 66 (86.9 %) cases and for the remaining 10 (13.1 %) Eusol-C was used. The median donor ECD was 3,021 cells/mm^2^ (interquartile range: 2,740–3,205), which is relatively high. This likely reflects a combination of surgeon preference for selecting higher-ECD grafts when available and donor pool quality, as all tissues were sourced from EBAA-accredited eye banks.

The causes of death and chronic comorbidities of the donors are provided in Supplementary Tables S1 and S2, respectively. Coronary artery disease and chronic obstructive pulmonary disease were the most common causes of death. Hypertension was the most prevalent chronic condition, followed by hyperlipidemia. Malignancies were observed in 11 donors, most commonly prostate cancer.

Descriptive statistics for corneal and graft thickness throughout the follow-up period are presented in Table 2. CCT for all cases showed a lowest mean value of 535 μm (SD = 64 μm) at 6 months postoperatively and a highest mean value of 789 μm (SD = 128 μm) recorded four hours after surgery (day 0). For CGT, the mean value ranged from 81 μm (SD = 27 μm) at 9 months to 170 μm (SD = 69 μm) on day 0.

**Fig. 1..**
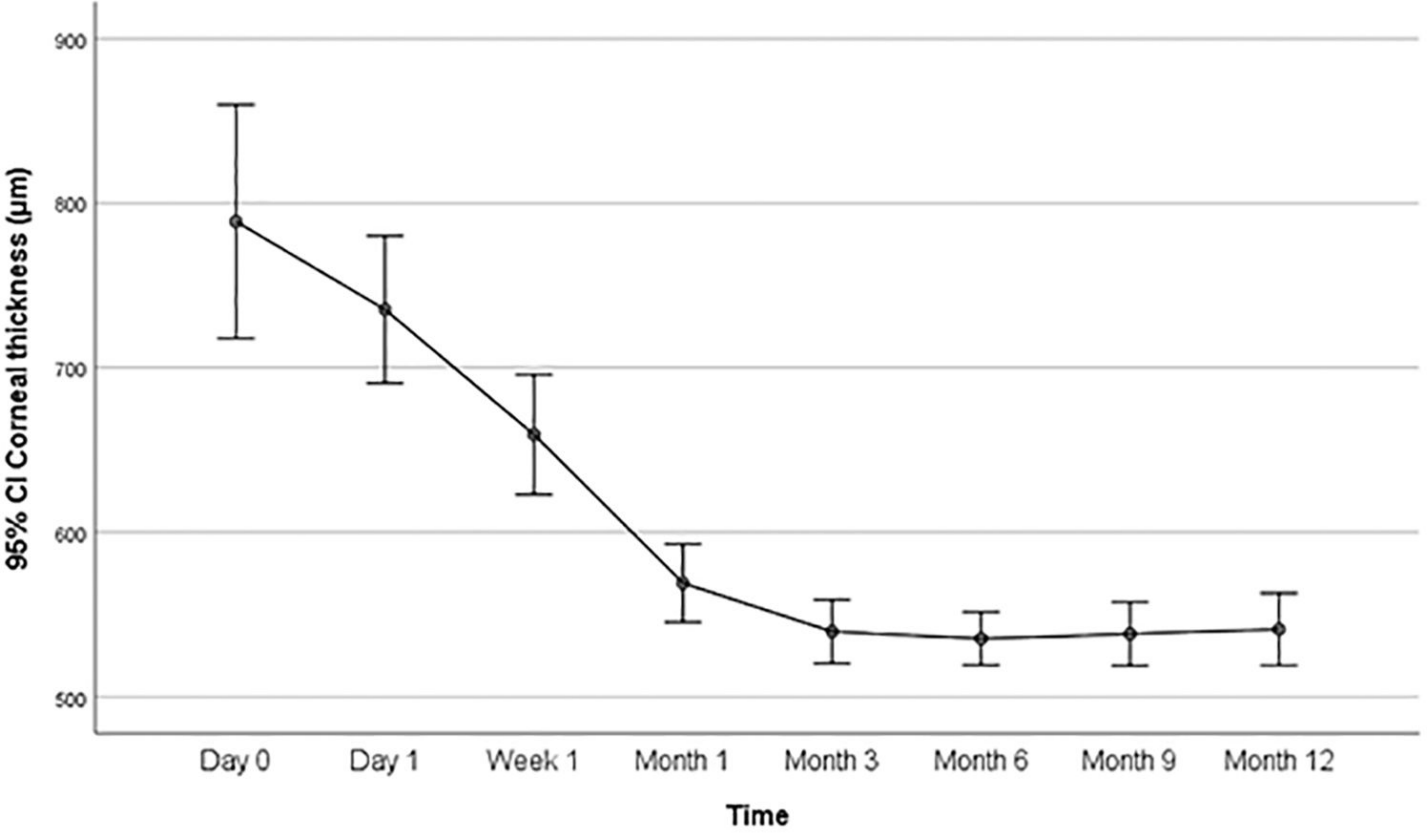
Recipient post-operative central corneal thickness (CCT) over time.

**Fig. 2..**
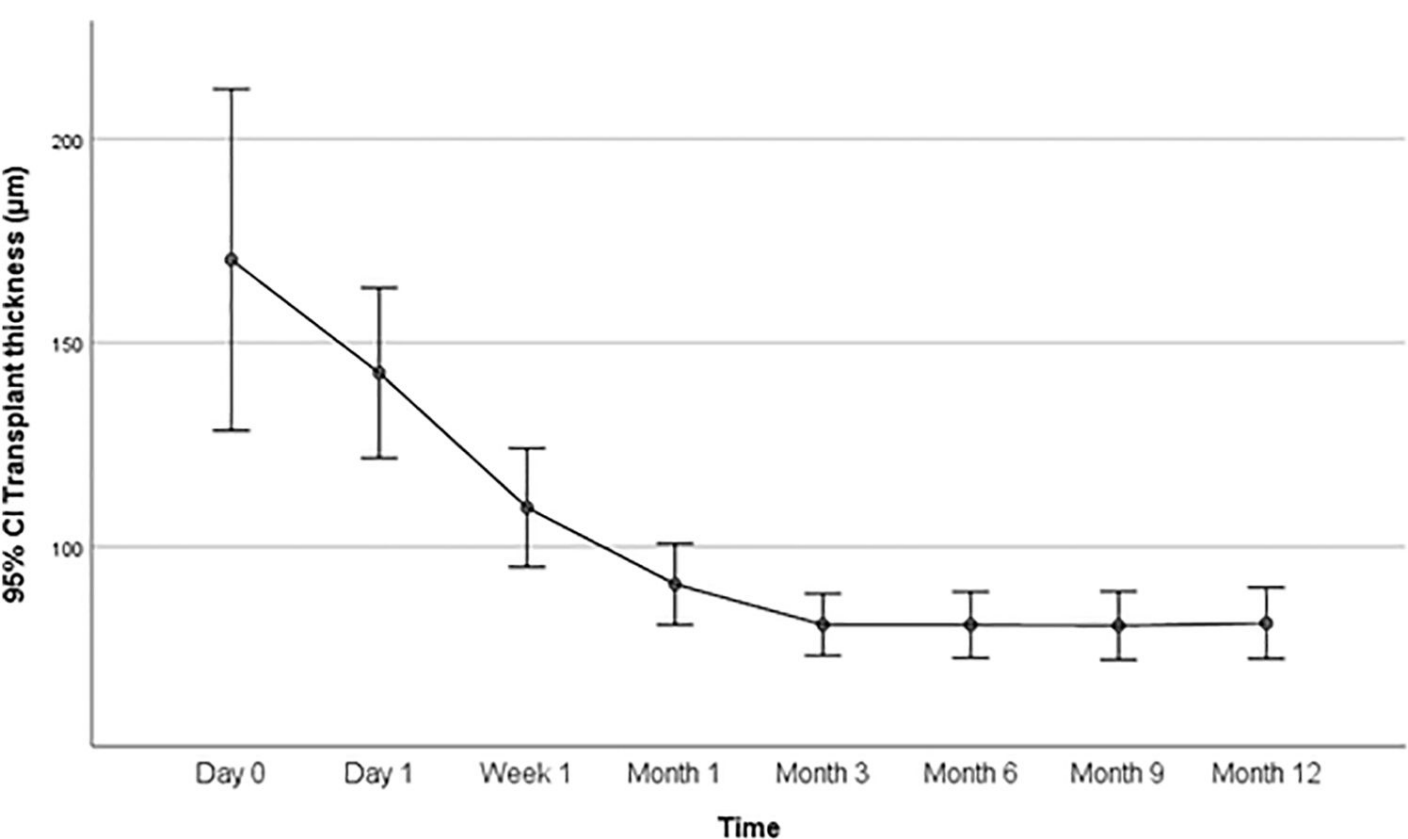
Recipient post-operative DSAEK central graft thickness (CGT) over time.

Fig. 1 illustrates the changes in recipient post-operative CCT throughout the follow-up period for all cases, while Fig. 2 shows the changes in recipient post-operative CGT for DSAEK cases only. As demonstrated in these Fig.s, both recipient post-operative CCT and DSAEK CGT decreased significantly up to 3 months post-surgery and subsequently stabilized through the 12-month follow-up period. This observation is further supported by the findings from the mixed linear regression models. Table 3 presents the results for recipient post-operative CCT, showing a significant decrease up to the third month (β = −1.73 μm/day, SE = 0.12; p < 0.001), followed by no significant change during the remaining follow-up period (β = 0.063 μm/day, SE = 0.045; p = 0.2). Similarly, Table 4 provides the results for recipient post-operative DSAEK CGT, indicating a significant reduction up to the third month (β = −0.53 μm/day, SE = 0.05; p < 0.001), with no significant changes thereafter (β = 0.02 μm/day, SE = 0.02; p = 0.2). These findings remained robust after adjusting for all other sample characteristics (as described in detail in the statistical analysis section).

**Fig. 3..**
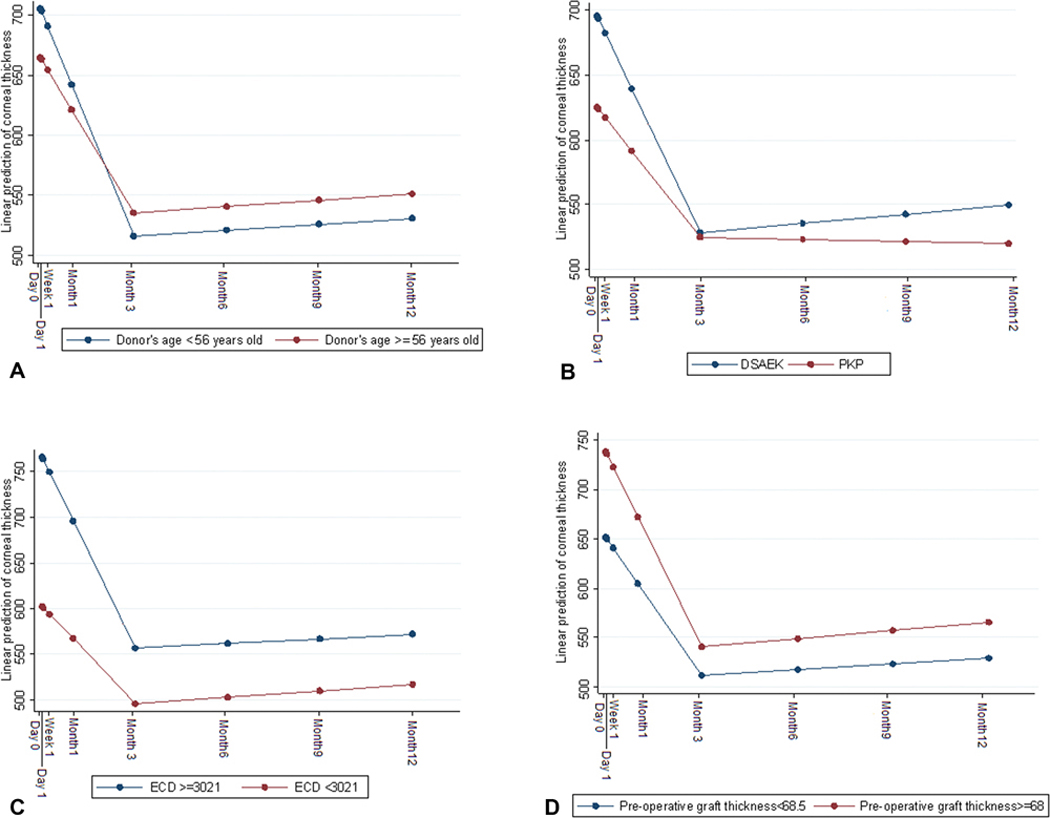
Model-predicted recipient post-operative central corneal thickness (CCT) over time, based on fixed effects from the mixed linear regression model. Stratified by donor age **(A)**, type of surgery **(B)**, donor graft endothelial cell density (ECD) **(C)**, and donor preoperative eye bank-measured DSAEK graft thickness **(D)**.

Recipients of grafts from younger donors (<56 years) had a significantly higher rate of corneal clearance during the first three post-operative months (β = 0.67 μm/day, SE = 0.23; p = 0.004; Table 3) (Fig. 3A). By the third month, corneal thickness was comparable between groups, with mean values of 531 μm (SD = 87) for donors aged <56 years and 549 μm (SD = 78) for donors aged ≥56 years (p = 0.4). Corneal thickness remained stable beyond the third month in both adjusting for recipient preoperative CCT, donor graft ECD, type of surgery, recipient age, preoperative diagnosis, donor diabetes status, DPT, death-to-surgery time, and cut-to-surgery time. Interpretations of all CCT-related regression outputs are provided in Supplementary Tables S3 and S4.

A significantly lower rate of corneal deturgescence was observed in PKP cases compared to DSAEK cases during the first three months, as indicated by a positive interaction term between time and surgical technique (β =0.74 μm/day, SE =0.32; p =0.021; Table 3) (Fig. 3B). This positive interaction coefficient suggests that the decrease in CCT over time was less steep for PKP, indicating slower clearance. By the third month, however, mean corneal thickness values were similar between the two groups; 543 μm (SD = 92) for DSAEK and 536 μm (SD = 41) for PKP (p = 0.8). After adjusting for all other covariates, the interaction term remained positive and approached statistical significance (β = 0.66 μm/day, SE = 0.39; p = 0.085), suggesting a persistent trend toward slower deturgescence in PKP eyes. All findings regarding the type of surgery remained significant after adjusting for recipient preoperative CCT, donor graft ECD, type of surgery, recipient age, preoperative diagnosis, donor diabetes status, DPT, death-to-surgery time, and cut-to-surgery time.

In cases with donor ECD ≥3,021 cells/mm^2^, the rate of corneal clearance during the first three months was significantly higher (β = −1.14 μm/day, SE = 0.21; p = 0.001; Table 3) (Fig. 3C). This effect persisted after adjusting for all other covariates (i.e. recipient preoperative CCT, type of surgery, recipient age, preoperative diagnosis, donor diabetes status, DPT, death-to-surgery time, and cut-to-surgery time). Moreover, in DSAEK cases donor graft thickness played a role in corneal clearance. Cases with eye bank-measured donor graft thickness ≥ 68.5 μm exhibited significantly greater corneal thickness on day 0 (β = 86.21, SE = 31.25; p = 0.006; Table 3), though by the third month, corneal thickness was comparable between groups, with mean values of 529 μm (SD = 94) for grafts < 68.5 μm and 555 μm (SD = 88) for grafts ≥ 68.5 μm (p = 0.3) (Fig. 3D). The rate of corneal clearance during the first three months was significantly higher in cases with thicker grafts (β = −0.64 μm/day, SE = 0.28; p = 0.022; Table 3). The findings regarding eye bank-measured donor graft thickness remained significant after adjusting for recipient preoperative CCT, donor graft ECD, type of surgery, recipient age, preoperative diagnosis, donor diabetes status, DPT, death-to-surgery time and cut-to-surgery time.

**Fig. 4..**
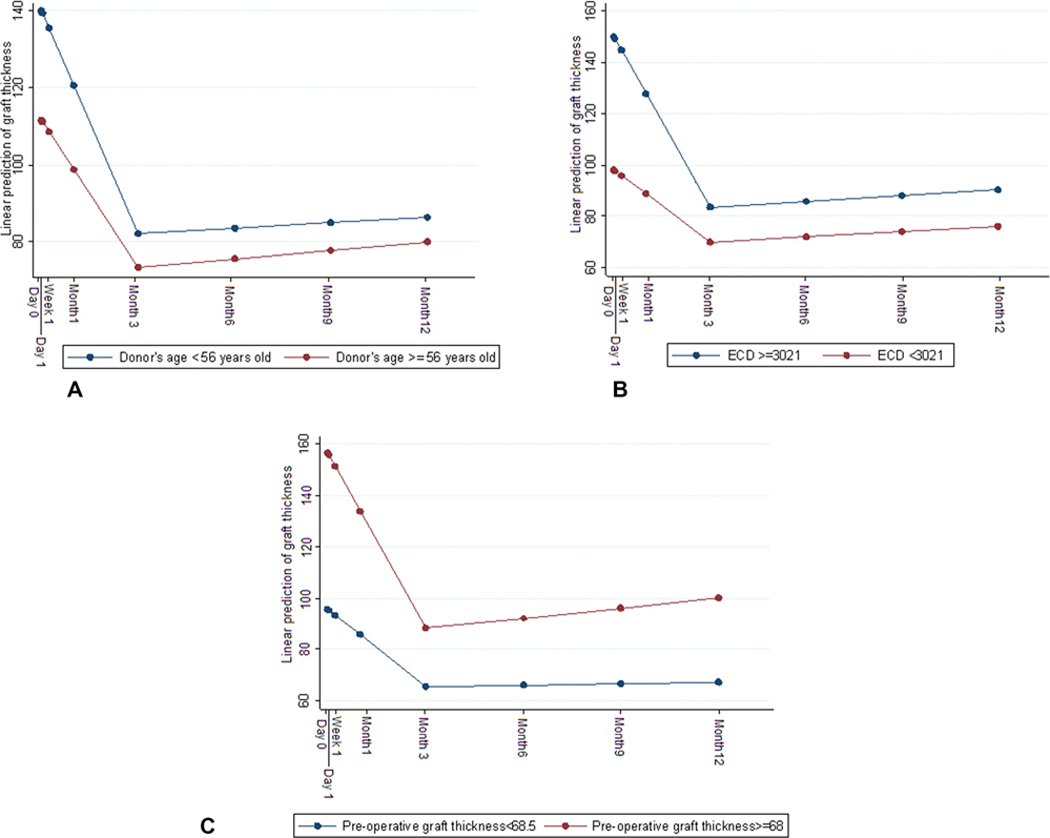
Model-predicted recipient post-operative DSAEK central graft thickness (CGT) over time, based on fixed effects from the mixed linear regression model. Stratified by donor age **(A)**, donor graft endothelial cell density (ECD) **(B)**, and donor preoperative eye bank-measured DSAEK graft thickness **(C)**.

Table 4 summarizes the results of mixed linear models for recipient post-operative CGT in DSAEK cases. The interaction effects during the first three months indicated that the rate of graft deturgescence was significantly slower when the donor was aged ≥56 years [interaction term β = 0.22 μm/day, SE = 0.10; p = 0.030] (Fig. 4A) and significantly faster in cases with donor ECD ≥3,021 cells/mm^2^ [interaction term β = −0.42 μm/day, SE = 0.09; p < 0.001] (Fig. 4B). These opposing directions of effect reflect how each variable modifies the slope of CGT reduction over time, with positive β values indicating slower clearance and negative values indicating accelerated clearance. These findings remained robust after adjusting for pre-operative eye bank-measured donor graft thickness, type of surgery, recipient age, preoperative diagnosis, donor diabetes status, DPT, death-to-surgery time, and cut-to-surgery time.

Donor preoperative graft thickness, as measured by the eye bank, was significantly associated with both baseline graft thickness and the rate of early post-operative clearance. Specifically, eyes receiving grafts with thickness ≥ 68.5 μm, had significantly higher CGT on day 0 [β = 60.77, SE = 9.31; p < 0.001; Table 4], as expected. Notably, these grafts also exhibited a significantly faster rate of clearance over the first three months [interaction term: [β = −0.42 μm/day, SE = 0.09; p < 0.001; Table 4]. At three months, CGT remained higher in this group (mean: 94 μm vs. 69 μm; p < 0.001). These associations remained robust after adjusting for relevant donor, surgical, and recipient characteristics. Interpretations of all CGT-related results are detailed in Supplementary Tables S5 and S6.

Finally, our analysis showed that both corneal and graft clearance rates over time were not significantly associated with recipient gender, recipient age, recipient pre-operative diagnosis, recipient pre-operative corneal thickness, donor diabetes status, donor graft death-to-preservation time, donor graft death-to-surgery time, or donor graft cut-to-surgery time.

## Discussion

This study provides valuable insights into the factors influencing the rate of corneal and graft clearance after transplantation, leveraging the capabilities of AS-OCT imaging to assess graft dynamics over time. Donor, recipient, and surgical factors were found to play pivotal roles in determining graft clearance rates, with the first three months post-surgery emerging as a critical period for recovery and stabilization.

Although recovery plateau timing was one focus of this study, the use of AS-OCT was central to its design. Compared to ultrasound pachymetry, AS-OCT provides higher-resolution, non-contact imaging with improved repeatability and reduced operator dependence, making it particularly well-suited for longitudinal evaluation of graft structure and clearance. Unlike ultrasound pachymetry, AS-OCT enabled us to differentiate between host and graft tissue, quantify CGT, and capture high-resolution structural detail. These features were essential for evaluating layer-specific clearance and dynamic graft-host interactions over time.

Donor age emerged as a significant determinant of graft clearance rates. Recipients of grafts from younger donors (< 56 years) exhibited a significantly higher rate of corneal deturgescence during the first three months compared to those with older donors (≥ 56 years). This effect persisted even after adjusting for other covariates, suggesting that younger donor tissue may facilitate faster post-operative recovery. The observed slower clearance in cases with older donors aligns with previous reports associating advanced donor age with reduced graft survival at 10 years after PKP in the Cornea Donor Study.^12,13^ On the other hand, in the Cornea Preservation Time Study older donor tissue had minimal impact on graft success following DSAEK surgery at 3 years post-operatively.^3,14^

While our analysis confirmed that younger donor age is significantly associated with faster graft clearance, identifying a specific age threshold for earlier recovery remains challenging. We explored multiple donor age cut-offs using quartile and tertile splits and compared model performance via the Akaike Information Criterion (AIC). Although the model based on the lowest age quartile yielded the best AIC, the corresponding β estimate was not significantly different from that obtained using the median split. These findings suggest that while donor age exerts a continuous influence on graft deturgescence, a sharp inflection point may not be easily defined. Future studies with larger cohorts and time-to-event endpoints may be better positioned to establish clinically actionable thresholds.

Donor ECD emerged as another critical determinant of graft outcomes. Cases with higher donor ECD (≥3,021 cells/mm^2^) demonstrated significantly faster rates of corneal clearance during the first three months, reflecting the importance of endothelial cell quality in graft function. This finding reinforces previous studies that linked higher ECD with reduced endothelial cell loss in PKP and DSAEK. Specifically, in the Cornea Donor Study, higher donor ECD correlated with reduced endothelial cell loss 10 years after PKP,^12,13^ while in the Cornea Preservation Time Study lower screening ECD was associated with lower graft ECD at 3 years after DSAEK.^3,14^

Although our findings suggest that higher donor endothelial cell density is associated with faster early graft clearance, we found no existing studies that specifically examine the impact of ECD on the rate of edema resolution in the immediate postoperative period. Most available literature centers on long-term outcomes, such as graft survival and cumulative endothelial cell loss. ^3,12,15–17^ Recent studies have highlighted that the biological quality of donor endothelial cells may play a pivotal role in graft outcomes, beyond traditional metrics such as donor ECD and age. Specifically, Kitazawa et al. demonstrated that donor corneas enriched with mature-differentiated corneal endothelial cells - characterized by surface markers CD166+, CD44^−/dull, CD24−, and CD105− - had significantly better long-term ECD retention and graft survival compared to those with lower maturity cells. ^18,19^ These findings suggest that standard screening parameters may overlook functionally compromised or senescent endothelial cells, and support a future role for biological maturity markers or metabolic assessments in donor tissue evaluation. Integrating such biomarkers into routine eye banking practices may further optimize graft longevity.

The surgical technique also significantly influenced graft clearance rates. DSAEK cases demonstrated faster rates of corneal deturgescence compared to PKP cases during the first three months post-operatively. By the third month, corneal thickness levels were comparable across techniques, but the initial advantage of DSAEK may reflect the procedural efficiency and reduced trauma associated with partial-thickness transplants. These findings support the growing preference for DSAEK and DMEK in clinical practice.

Post-operative corneal and graft thickness remained stable after three months, regardless of donor, recipient or surgical factors. This suggests that the first three months represent a critical period for post-operative recovery and graft clearance, after which long-term outcomes are less influenced by the initial donor or surgical characteristics. Stability beyond this period also underscores the importance of effective management during the early post-operative phase to ensure optimal long-term graft function.

The observation that thicker donor grafts were associated with faster early deturgescence may reflect inherent stromal hydration differences. Thicker preoperative grafts may retain more fluid or represent tissue less affected by pre-cut dehydration, enabling more pronounced early deturgescence. Additionally, the preserved stromal structure and endothelial integrity in these grafts may facilitate more efficient fluid pumping in the postoperative period. These findings align with the hypothesis that early graft dynamics are not solely dependent on baseline thickness but also on tissue responsiveness and hydration gradients.

Interestingly, factors such as recipient gender, age, pre-operative CCT, pre-operative diagnosis, donor diabetes status, and preservation-related times (DPT, death-to-surgery, and cut-to-surgery) were not significantly associated with graft clearance rates. These findings suggest that while these factors may play a role in specific contexts, they do not universally impact early post-operative outcomes.

While recipient age did not show a significant direct association with graft clearance rates, it remains a clinically relevant factor given its potential impact on post-operative compliance and outcomes. In the Cornea Donor Study, Sugar et al (2014) found an association between recipient age and graft failure after PKP, showing higher failure rates in recipients aged 70 or older compared to those younger than 60 (29 % vs 19 %; p value = 0.04).^15^

Recipient pre-operative CCT has been studied as a predictor of graft success in DMEK with conflicting results. Brockmann et al. found that thinner pre-operative CCT (<625 μm) correlated with better visual outcomes.^20^ However, other studies indicate that post-operative, rather than pre-operative, CCT is a stronger predictor of visual outcomes at 12 months.^21^ In our study, pre-operative CCT did not significantly influence graft clearance rates.

The impact of pre-operative diagnosis has been well-documented. Recipients with Fuchs endothelial corneal dystrophy (FECD) consistently show better long-term outcomes compared to those with pseudophakic or aphakic corneal edema (PACE), regardless of the type of keratoplasty. This has been demonstrated in DSAEK,^14^ DMEK ,^11^ and PKP, where the 10-year cumulative graft failure rate for PACE (37 %) is significantly higher than for FECD (20 %).^15^ However, in our analysis, pre-operative diagnosis did not significantly influence early graft clearance.

Donor diabetes status presented varied implications in previous studies. While it increases the risk of DMEK tissue preparation failure, it does not significantly affect long-term graft survival, adherence, or the need for air reinjection.^8^ For DSAEK, donor diabetes has been associated with reduced post-operative ECD at three years,^3^ but no significant impact on ECD loss or graft failure was observed for PKP grafts over 10 years.^16,22^ In our study, donor diabetes did not significantly affect early graft clearance.

Preservation times, including DPT and death-to-surgery time, also showed mixed findings in the literature. Longer death-to-surgery intervals are linked to greater endothelial cell loss and lower graft success at three years post-DSAEK.^9,23^ However, death-to-surgery times up to five days did not significantly impact PKP survival or endothelial cell density over five years.^17,24^ Conflicting results were also observed for DPT, with some studies reporting no significant effects on endothelial cell density,^25,26^ while others identified increased endothelial cell loss with DPTs exceeding 10 hours.^27^ Refrigeration of donor tissue has been shown to mitigate the risks associated with longer DPTs.^28^ In our study, preservation times had no significant impact on early graft clearance. Overall, while the aforementioned factors may influence certain aspects of graft health or long-term outcomes, their role in early post-operative graft clearance appears limited.

The results of this study have several clinical implications. First, they emphasize the importance of donor tissue quality, particularly in terms of age, endothelial cell density, and graft thickness, in achieving faster post-operative recovery. Second, they highlight the advantages of DSAEK and DMEK in facilitating faster recovery compared to PKP. Third, they underscore the need for careful monitoring and management during the first three months post-operatively, which is the most dynamic period for graft clearance and stabilization. Fourth, the use of serial AS-OCT imaging highlights its potential as a non-invasive tool for monitoring graft health, enabling early intervention in cases of graft dysfunction and improving long-term success rates. Lastly, while the long-term functional impact of faster deturgescence remains to be fully established, our findings may have particular clinical relevance in scenarios where early visual recovery is important - such as in monocular patients, those requiring second-eye surgery within a short interval, or individuals with high occupational visual demands. We do not propose universal prioritization of younger donors or higher ECD tissues; rather, we suggest these factors could guide selective tissue allocation in cases where accelerated postoperative recovery is clinically meaningful.

While our study provides valuable insights, it is important to acknowledge its limitations. As with many studies in this field, the heterogeneity of corneal pathologies and surgical techniques can make it challenging to draw broad conclusions. Additionally, the small sample size and small number of DMEK grafts included in this analysis may affect the generalizability of our findings. Furthermore, the exclusion of grafts with postoperative complications, including early failure and rejection, introduces a potential selection bias. While this was necessary to focus on grafts with evaluable deturgescence patterns, it may limit the generalizability of our findings to broader clinical populations where such complications are part of the postoperative spectrum. Future studies should focus on validating these findings across larger and more diverse cohorts. Although our study demonstrated significant associations between donor factors and the rate of reduction in CCT and CGT, we did not formally evaluate the relationship between these thickness parameters and clinical graft clarity or visual acuity. While CCT and CGT are valuable structural markers of deturgescence, they may only partially reflect patient-perceived visual quality. Corneal clarity - particularly when measured by densitometry - may be more predictive of visual acuity than thickness alone.^29^ As such, pachymetric improvement may represent structural recovery but not necessarily translate directly to functional visual gains. Future studies that correlate serial pachymetric trends with visual acuity and optical quality measures will be essential to validate these markers as surrogates for patient-centered outcomes, thereby enhancing the translational relevance of serial AS-OCT monitoring. It is worth noting that in this study several continuous variables were dichotomized to improve interpretability and visualization. While this may have reduced statistical power, sensitivity analyses using continuous forms produced similar results. Finally, the integration of machine learning algorithms with AS-OCT imaging data could potentially enhance our ability to predict graft outcomes and tailor post-operative management strategies.

In conclusion, this study highlights the critical role of targeted donor selection and early post-operative management in optimizing long-term corneal graft success. Factors such as donor age and endothelial cell density significantly influence the rate of graft clearance, underscoring the need for careful evaluation during the donor selection process. The use of serial AS-OCT imaging in our study demonstrates its value as an advanced, non-invasive tool for monitoring and assessing graft health over time. AS-OCT’s ability to provide detailed, repeatable measurements has significant potential for improving post-operative care in clinical practice and refining graft assessment protocols in future research.

## Supplementary Material

1

2

3

4

Supplementary material associated with this article can be found, in the online version, at doi:10.1016/j.ajoint.2025.100147.

## Figures and Tables

**Table 1.** Sample characteristics for transplant recipients and donor grafts.

*n=76*		n	%
Recipient gender	Female	37	48.7
	Male	39	51.3
Recipient pre-operative diagnosis	PACE	30	41.7
	FECD	28	38.9
	Failed graft	14	19.4
Type of surgery	DMEK	3	3.9
	DSAEK	57	75.0
	PKP	16	21.1
Donor diabetes status	No	62	82.7
Donor gender	Yes	13	17.3
Donor preservation media	Female	27	35.5
	Male	49	64.5
	Optisol-GS	66	86.9
	Eusol-C	10	13.1
		Median	IQR
Recipient age		74.0	66.0 – 82.5
Recipient pre-operative corneal thickness (CCT, μm)		706.5	648.0 – 828.0
Donor age		56.0	42.0 – 67.0
Donor graft endothelial cell density (ECD, cells/mm^2^)		3021.0	2740.0 – 3205.0
Donor graft death-to-preservation time (DPT, minutes)		984.0	685.0 – 1238.0
Donor eye bank-measured graft thickness (μm)^1^		68.5	60.0 – 83.5
Donor graft death-to-surgery time (hours)		196.0	168.0 – 218.0
Donor graft cut-to-surgery time (hours)^1^		81.0	63.0 – 94.0
Recipient pre-operative vision (logMAR)		1.25	1.00 – 2.30
Recipient vision at last follow-up (logMAR)		0.40	0.23 – 0.50

1. only for DSAEK cases

Abbreviations: PACE, pseudophakic or aphakic corneal edema; FECD, Fuchs endothelial corneal dystrophy; DMEK, Descemet membrane endothelial keratoplasty; DSAEK, Descemet-stripping automated endothelial keratoplasty; PKP, penetrating keratoplasty.

**Table 2.** Recipient post-operative corneal and graft thickness over time.

		n	Mean (SD)
Central corneal thickness (μm)	Day 0	15	789 (128)
	1 day	54	735 (164)
	1 week	64	659 (146)
	1 month	71	569 (100)
	3 months	72	540 (82)
	6 months	62	535 (64)
	9 months	52	538 (70)
	12 months	44	541 (72)
Central graft thickness (μm)^1^	Day 0	13	170 (69)
	1 day	48	143 (72)
	1 week	50	110 (51)
	1 month	54	91 (37)
	3 months	54	81 (28)
	6 months	47	81 (28)
	9 months	42	81 (27)
	12 months	37	81 (26)

1. Only for DSAEK cases.

**Table 3.** Mixed linear regression results for recipient post-operative central corneal thickness (CCT) as the dependent variable.

*Dependent variable:* Recipient post-operative central corneal thickness (μm)	Main effect	
	β (SE)^+^	P
*Constant*	681.23 (12.71)	<0.001
Time (≤3 months)	−1.73 (0.12)	<0.001
Time (>3 months)	0.063 (0.045)	0.156
Recipient age	0.29 (1.01)	0.776
Time (≤3 months)	−1.74 (0.12)	<0.001
Time (>3 months)	0.06 (0.04)	0.157
Type of surgery (PKP vs DSAEK)	−70.10 (32.93)	0.033
Time (≤3 months)	−1.86 (0.14)	<0.001
Time (>3 months)	0.08 (0.05)	0.114
Interaction Surgery * Time (≤3 months)	0.74 (0.32)	0.021
Interaction Surgery * Time (>3 months)	−0.10 (0.13)	0.463
Recipient pre-operative diagnosis PACE vs Failed graft	61.47 (33.75)	0.069
FECD vs Failed graft	33.53 (34.03)	0.325
Time (≤3 months)	−1.77 (0.12)	<0.001
Time (>3 months)	0.07 (0.13)	0.126
Recipient pre-operative CCT (μm)	0.09 (0.09)	0.325
Time (≤3 months)	−1.74 (0.13)	<0.001
Time (>3 months)	0.07 (0.05)	0.145
Donor age (≥56 vs <56) ^1^	−40.80 (19.77)	0.045
Time (≤3 months)	−2.10 (0.17)	<0.001
Time (>3 months)	0.05 (0.07)	0.455
Interaction Donor age * Time (≤3 months)	0.67 (0.23)	0.004
Interaction Donor age * Time (>3 months)	0.004 (0.09)	0.968
Donor diabetes status (yes vs no)	−0.42 (31.85)	0.989
Time (≤3 months)	−1.74 (0.12)	<0.001
Time (>3 months)	0.06 (0.05)	0.168
Donor graft ECD (≥3021 vs <3021)^1^	163.10 (20.16)	<0.001
Time (≤3 months)	−1.19 (0.15)	<0.001
Time (>3 months)	0.08 (0.06)	0.226
Interaction ECD * Time (≤3 months)	−1.14 (0.21)	<0.001
Interaction ECD * Time (>3 months)	−0.02 (0.09)	0.813
Donor preoperative eye bank-measured graft thickness (μm) (≥68.5 vs <68.5)^1,2^	86.21 (31.25)	0.006
Time (≤3 months)	−1.56 (0.20)	<0.001
Time (>3 months)	0.06 (0.07)	0.371
Interaction Donor graft thickness * Time (≤3 months)	−0.64 (0.28)	0.022
Interaction Donor graft thickness * Time (>3 months)	0.03 (0.10)	0.775
Donor graft death-to-preservation time (minutes)	0.05 (0.04)	0.177
Time (≤3 months)	−1.74 (0.12)	<0.001
Time (>3 months)	0.06 (0.05)	0.166
Donor graft death-to-surgery time (hours)	0.53 (0.34)	0.123
Time (≤3 months)	−1.74 (0.12)	<0.001
Time (>3 months)	0.06 (0.05)	0.170
Donor graft cut-to-surgery time (hours)	−0.36 (0.41)	0.375
Time (≤3 months)	−1.84 (0.14)	<0.001
Time (>3 months)	0.08 (0.05)	0.111

Note: The *Constant* represents the estimated CCT in μm at baseline (day 0) for the reference group. Each covariate row displays the main effect (μm), representing its association with baseline CCT. Where statistically significant, interaction terms with time are included. Time is modeled using a spline with a knot at 3 months (≤3 months and >3 months). Interaction coefficients represent the difference in rate of CCT change (μm/day) across subgroups. All interaction terms were tested; only statistically significant interactions are reported for clarity.

+. regression coefficient (Standard Error)

1. categorized according to median value

2. only for DSAEK cases *Abbreviations: PACE, pseudophakic or aphakic corneal edema; FECD, Fuchs endothelial corneal dystrophy; DSAEK, Descemet-stripping automated endothelial keratoplasty; PKP, penetrating keratoplasty; ECD, endothelial cell density*.

**Table 4.** Mixed linear regression results for recipient post-operative central graft thickness (CGT) in DSAEK cases as the dependent variable.

*Dependent variable:* Recipient post-operative central corneal thickness (μm)	Main effect	
	β (SE)^+^	P
*Constant*	124.77 (6.05)	<0.001
Time (≤3 months)	−0.53 (0.05)	<0.001
Time (>3 months)	0.02 (0.02)	0.228
Recipient age	−0.71 (0.54)	0.188
Time (≤3 months)	−0.53 (0.05)	<0.001
Time (>3 months)	0.02 (0.02)	0.229
Recipient pre-operative diagnosis PACE vs Failed graft	11.32 (21.31)	0.595
FECD vs Failed graft	13.06 (21.41)	0.542
Time (≤3 months)	−0.53 (0.05)	<0.001
Time (>3 months)	0.02 (0.02)	0.230
Reciepient pre-operative CCT (μm)	0.004 (0.05)	0.936
Time (≤3 months)	−0.54 (0.05)	<0.001
Time (>3 months)	0.03 (0.02)	0.195
Donor age (≥56 vs <56) ^1^	−28.44 (11.48)	0.013
Time (≤3 months)	−0.64 (0.07)	<0.001
Time (>3 months)	0.02 (0.03)	0.612
Interaction Donor age * Time (≤3 months)	0.22 (0.10)	0.030
Interaction Donor’s age * Time (>3 months)	0.01 (0.04)	0.819
Donor diabetes status (yes vs no)	−14.97 (14.63)	0.306
Time (≤3 months)	−0.53 (0.05)	<0.001
Time (>3 months)	0.02 (0.02)	0.227
Donor graft ECD (≥3021 vs <3021) ^1^	51.80 (10.01)	<0.001
Time (≤3 months)	−0.31 (0.07)	<0.001
Time (>3 months)	0.02 (0.03)	0.400
Interaction ECD * Time (≤3 months)	−0.42 (0.09)	<0.001
Interaction ECD * Time (>3 months)	0.002 (0.04)	0.957
Donor preoperative eye bank-measured graft thickness (μm) (≥68.5 vs <68.5) ^1^	60.77 (9.31)	<0.001
Time (≤3 months)	−0.34 (0.06)	<0.001
Time (>3 months)	0.01 (0.03)	0.828
Interaction Donor graft thickness * Time (≤3 months)	−0.42 (0.09)	<0.001
Interaction Donor graft thickness * Time (>3 months)	0.04 (0.04)	0.329
Donor graft death-to-preservation time (minutes)	0.01 (0.02)	0.742
Time (≤3 months)	−0.53 (0.05)	<0.001
Time (>3 months)	0.02 (0.02)	0.228
Donor graf death-to-surgery time (hours)	0.30 (0.20)	0.134
Time (≤3 months)	−0.53 (0.05)	<0.001
Time (>3 months)	0.02 (0.02)	0.227
Donor graft cut-to-surgery time (hours)	−0.08 (0.15)	0.584
Time (≤3 months)	−0.53 (0.05)	<0.001
Time (>3 months)	0.02 (0.02)	0.228

Note. Only DSAEK cases are included in this analysis. The *Constant* represents the estimated central graft thickness (CGT) in μm at baseline (day 0) for the reference group. Each covariate row displays the main effect (μm) and, when significant, time-covariate interaction effects. Time was modeled using a spline with a knot at 3 months (≤3 months and >3 months). Interaction terms represent differences in rate of CGT change (μm/day). Only statistically significant interactions are shown.

+. regression coefficient (Standard Error)

1. categorized according to median value

Abbreviations: PACE, pseudophakic or aphakic corneal edema; FECD, Fuchs endothelial corneal dystrophy; DSAEK, Descemet-stripping automated endothelial keratoplasty; PKP, penetrating keratoplasty; ECD, endothelial cell density.

## Data Availability

All datasets generated during and/or analyzed during the current study are available from the corresponding author upon reasonable request.

## References

[1] Romano V, Passaro ML, Ruzza A, Quality assurance in corneal transplants: donor cornea assessment and oversight. Surv Ophthalmol. 2024;69(3):465–482. 10.1016/j.survophthal.2023.12.002. 38199504

[2] O’Brien RC, Ishwaran H, Szczotka-Flynn LB, Lass JH. Cornea preservation time Study (CPTS) group. Random survival forests analysis of intraoperative complications as predictors of descemet stripping automated endothelial keratoplasty graft failure in the Cornea preservation time study. JAMA Ophthalmol. 2021;139(2):191. 10.1001/jamaophthalmol.2020.5743. 33355637 PMC7758826

[3] Lass JH, Benetz BA, Patel SV, Donor, recipient, and operative factors associated with increased endothelial cell loss in the cornea preservation time study. JAMA Ophthalmol. 2019;137(2):185. 10.1001/jamaophthalmol.2018.5669. 30422157 PMC6439830

[4] Dunker SL, Armitage WJ, Armitage M, Practice patterns of corneal transplantation in Europe: first report by the European Cornea and Cell Transplantation Registry. J Cataract Refract Surg. 2021;47(7):865–869. 10.1097/j.jcrs.0000000000000574. 33577274

[5] Brown DA, Martinez Guasch F, Li A, Sunshine SB. Surgical advancements in corneal transplantation. Curr Surg Rep. 2022;10(12):246–254. 10.1007/s40137-022-00335-8.

[6] Ang M, Baskaran M, Werkmeister RM, Anterior segment optical coherence tomography. Progr Retinal Eye Res. 2018;66:132–156. 10.1016/j.preteyeres.2018.04.002. 29635068

[7] Neokleous A, Michail N, Herodotou F, Long-term monitoring of corneal grafts via anterior segment OCT pachymetry maps. Ophthalmol Sci. 2025;5(4), 100724. 10.1016/j.xops.2025.100724. PMC11964645 40182982

[8] Price MO, Lisek M, Feng MT, Price FW. Effect of donor and recipient diabetes status on descemet membrane endothelial keratoplasty adherence and survival. Cornea. 2017;36(10):1184–1188. 10.1097/ICO.0000000000001305. 28749899

[9] Rosenwasser GO, Szczotka-Flynn LB, Ayala AR, Effect of cornea preservation time on success of descemet stripping automated endothelial keratoplasty: A randomized clinical trial. JAMA Ophthalmol. 2017;135(12):1401. 10.1001/jamaophthalmol.2017.4989. 29127431 PMC6583547

[10] Potapenko IO, Samolov B, Armitage MC, Byström B, Hjortdal J. Donor endothelial cell count does not correlate with descemet stripping automated endothelial keratoplasty transplant survival after 2 years of follow-up. Cornea. 2017;36(6): 649–654. 10.1097/ICO.0000000000001189. 28410357

[11] Baydoun L, Ham L, Borderie V, Endothelial survival after descemet membrane Endothelial keratoplasty: effect of surgical indication and graft adherence status. JAMA Ophthalmol. 2015;133(11):1277. 10.1001/jamaophthalmol.2015.3064. 26355238

[12] Lass JH, Benetz BA, Gal RL, Donor age and factors related to endothelial cell loss 10 years after penetrating keratoplasty. Ophthalmology. 2013;120(12): 2428–2435. 10.1016/j.ophtha.2013.08.044. 24246826 PMC3835371

[13] Mannis MJ, Holland EJ, Gal RL, The effect of donor age on penetrating keratoplasty for endothelial disease. Ophthalmology. 2013;120(12):2419–2427. 10.1016/j.ophtha.2013.08.026. 24246825 PMC3885822

[14] Terry MA, Aldave AJ, Szczotka-Flynn LB, Donor, recipient, and operative factors associated with graft success in the cornea preservation time study. Ophthalmology. 2018;125(11):1700–1709. 10.1016/j.ophtha.2018.08.002. 30098353 PMC6196643

[15] Sugar A, Gal RL, Kollman C, Factors associated with corneal graft survival in the cornea donor study. JAMA Ophthalmol. 2015;133(3):246. 10.1001/jamaophthalmol.2014.3923. 25322173 PMC4394864

[16] Lass JH, Riddlesworth TD, Gal RL, The effect of donor diabetes history on graft failure and endothelial cell density 10 years after penetrating keratoplasty. Ophthalmology. 2015;122(3):448–456. 10.1016/j.ophtha.2014.09.012. 25439611 PMC4339512

[17] Lass JH. Baseline factors related to endothelial cell loss following penetrating keratoplasty. Arch Ophthalmol. 2011;129(9):1149. 10.1001/archophthalmol.2011.102. 21555600 PMC4186996

[18] Kitazawa K, Toda M, Ueno M, Donor corneal endothelial cell maturity and its impact on graft survival in Glaucoma patients undergoing corneal transplantation. Am J Ophthalmol. 2024;262:1–9. 10.1016/j.ajo.2024.01.033. 38307212

[19] Kitazawa K, Toda M, Ueno M, Uehara A, Sotozono C, Kinoshita S. The biologic character of donor corneal endothelial cells influences endothelial cell density post successful corneal transplantation. Ophthalmol Sci. 2023;3(2), 100239. 10.1016/j.xops.2022.100239. PMC9944567 36846106

[20] Brockmann T, Pilger D, Brockmann C, Maier AKB, Bertelmann E, Torun N. Predictive factors for clinical outcomes after primary Descemet’s membrane endothelial keratoplasty for Fuchs’ Endothelial dystrophy. Curr Eye Res. 2019;44(2): 147–153. 10.1080/02713683.2018.1538459. 30339062

[21] Moskwa R, Bloch F, Vermion JC, Postoperative, but not preoperative, central corneal thickness correlates with the postoperative visual outcomes of descemet membrane endothelial keratoplasty. Mimouni M, ed. PLoS ONE. 2023;18(3): e0282594. doi:10.1371/journal.pone.0282594. PMC9983850 36867645

[22] Vislisel JM, Liaboe CA, Wagoner MD, Graft survival of diabetic versus nondiabetic donor tissue after initial keratoplasty. Cornea. 2015;34(4):370–374. 10.1097/ICO.0000000000000378. 25642643

[23] Lass JH, Benetz BA, Verdier DD, Corneal endothelial cell loss 3 years after successful descemet stripping automated endothelial keratoplasty in the cornea preservation time study: A randomized clinical trial. JAMA Ophthalmol. 2017;135 (12):1394. 10.1001/jamaophthalmol.2017.4970. 29127432 PMC6583548

[24] Sugar J, Montoya M, Dontchev M, Donor risk factors for graft failure in the cornea Donor study. Cornea. 2009;28(9):981–985. 10.1097/ICO.0b013e3181a0a3e6. 19724216 PMC3124710

[25] Mohamed A, Chaurasia S, Garg P. Outcome of transplanted donor corneas with more than 6 h of death-to-preservation time. Indian J Ophthalmol. 2016;64(9):635. 10.4103/0301-4738.194338. 27853009 PMC5151151

[26] Sun MJ, Duong AT, Tran KD, Straiko MMW, Stoeger CG, Sales CS. Primary graft failure, infection, and endothelial cell density in corneal transplants with increased death-to-preservation time. Cornea. 2021;40(11):1462–1465. 10.1097/ICO.0000000000002697. 33734162 PMC8505130

[27] Parekh M, Salvalaio G, Ferrari S, Effect of postmortem interval on the graft endothelium during preservation and after transplantation for keratoconus. Cornea. 2013;32(6):842–846. 10.1097/ICO.0b013e318283c873. 23538616

[28] Patel D, Tandon R, Ganger A, Vij A, Lalwani S, Kumar A. Study of death to preservation time and its impact on utilisation of donor corneas. Trop Doct. 2017;47 (4):365–370. 10.1177/0049475517713406. 28610538

[29] Piotrowiak-Słupska I, Kałużny BJ, Malukiewicz G. Corneal optical densitometry in the evaluation of 2-year graft function following endothelial keratoplasty. JCM. 2023;12(4):1552. 10.3390/jcm12041552. 36836087 PMC9963363

