# Supplementary material for: Impact of recipient and donor factors on corneal graft clearance: insights from serial anterior segment optical coherence tomography
Source: AJO Int. Author manuscript; Available in PMC 2026 Apr 7. (PMC13052495; doi:10.1016/j.ajoint.2025.100147)
Supplement: 1 [file NIHMS2107585-supplement-1.docx]

**Supplementary Online Table S1.** Donor cause of death

| n=76 | N | % |
| --- | --- | --- |
| Cardiac shock | 1 | 1.3 |
| Trauma | 5 | 6.5 |
| Acute kidney injury | 1 | 1.3 |
| Coronary artery disease | 22 | 29 |
| Hanging | 2 | 2.7 |
| Electrocution | 2 | 2.7 |
| Cancer | 4 | 5.2 |
| End-stage liver disease | 6 | 7.8 |
| Gastrointestinal bleed | 1 | 1.3 |
| Aspiration | 1 | 1.3 |
| Pneumonia | 3 | 3.9 |
| Cerebrovascular accident | 1 | 1.3 |
| Chronic obstructive pulmonary disease | 8 | 10.5 |
| Abdominal aortic aneurysm | 1 | 1.3 |
| Cardiomyopathy | 2 | 2.7 |
| Overdose | 4 | 5.2 |
| Seizure | 2 | 2.7 |
| Chronic heart failure | 1 | 1.3 |
| Intracranial bleeding/Intracranial hemoorrhage | 6 | 7.8 |
| Anoxic brain injury | 1 | 1.3 |
| Multisystem organ failure | 2 | 2.7 |
