# Supplementary material for: Impact of recipient and donor factors on corneal graft clearance: insights from serial anterior segment optical coherence tomography
Source: AJO Int. Author manuscript; Available in PMC 2026 Apr 7. (PMC13052495; doi:10.1016/j.ajoint.2025.100147)
Supplement: 2 [file NIHMS2107585-supplement-2.docx]

**Supplementary Online Table S2.** Donor underlying chronic diseases

|  | N |
| --- | --- |
| Hypertension | 32 |
| Irritable bowel syndrome | 1 |
| Cancer | 11 |
| Hypothyroidism | 3 |
| Common variable immunodeficiency | 2 |
| Alpha-1 antitrypsin deficiency | 2 |
| Primary adrenocortical deficiency | 2 |
| Hyperlipidemia | 21 |
| Abdominal aortic aneurysm | 1 |
| Gout | 4 |
| Pulmonary hypertension | 2 |
| Shone’s syndrome | 2 |
| Mitral stenosis | 2 |
| Mitral regurgitation | 2 |
| Chronic heart failure | 9 |
| Asthma | 4 |
| Chronic obstructive pulmonary diseaase | 12 |
| Seizures | 4 |
| Ischemic cardiomyopathy | 5 |
| Diabetes mellitus | 15 |
| Mental disorders | 8 |
| Chronic kidney disease | 4 |
| Non-alcoholic steatohepatitis | 2 |
| Liver cirrhosis | 4 |
| Gastroesophageal reflux disease | 3 |
| Cerebrovascular accident | 7 |
| Coronary artery disease | 12 |
| Obesity | 1 |
| End-stage liver disease | 2 |
| Sinus venous thrombosis | 1 |
| Drug abuse | 4 |
| Smoking | 5 |
| Heart valve disease | 1 |
| Alcohol abuse | 4 |
| Ischemic colitis | 1 |
| Arthritis | 1 |
| Fatty liver | 2 |
| Subarachnoid hemorrhage/subdural hematoma | 1 |
| Cerebral palsy | 1 |
| Pulmonary fibrosis | 1 |
| Eczema | 1 |
| Pulmonary embolism | 1 |
| Deep vein thrombosis | 1 |
