# Supplementary material for: Impact of recipient and donor factors on corneal graft clearance: insights from serial anterior segment optical coherence tomography
Source: AJO Int. Author manuscript; Available in PMC 2026 Apr 7. (PMC13052495; doi:10.1016/j.ajoint.2025.100147)
Supplement: 3 [file NIHMS2107585-supplement-3.docx]

# Supplemental Appendix: Interpretation of Regression Model Outputs (Table 3)

## Supplementary Online Table S3. Interpretation of Main Effects (Table 3)

| Covariate | Interpretation |
| --- | --- |
| Recipient age | Each additional year of recipient age is associated with an average increase of 0.29 μm in baseline CCT, assuming other variables remain constant. |
| Type of surgery (PKP vs DSAEK) | Patients undergoing PKP have, on average, 70.10 μm lower baseline CCT compared to those undergoing DSAEK. |
| Recipient pre-operative diagnosis (PACE vs Failed graft) | Recipients with PACE have 61.47 μm higher baseline CCT compared to those with failed grafts. |
| Recipient pre-operative diagnosis (FECD vs Failed graft) | Recipients with FECD have 33.53 μm higher baseline CCT compared to those with failed grafts. |
| Recipient pre-operative CCT | Each additional μm in pre-operative CCT is associated with a 0.09 μm increase in baseline post-operative CCT. |
| Donor age (≥56 vs <56) | Recipients of grafts from donors aged ≥56 have, on average, 40.80 μm lower baseline CCT compared to those from younger donors. |
| Donor diabetes status (Yes vs No) | No meaningful difference in baseline CCT was observed between diabetic and non-diabetic donors (β = -0.42 μm, p = 0.989). |
| Donor graft ECD (≥3021 vs <3021) | Grafts with higher donor ECD (≥3021 cells/mm²) were associated with 163.10 μm higher baseline CCT compared to those with lower ECD. |
| Donor preoperative graft thickness (≥68.5 vs <68.5) | Grafts with preoperative thickness ≥68.5 μm had 86.21 μm higher baseline CCT. |
| Donor death-to-preservation time | Each additional minute from death to preservation is associated with a 0.05 μm increase in baseline CCT. |
| Donor death-to-surgery time | Each additional hour between donor death and surgery is associated with a 0.53 μm increase in baseline CCT. |
| Donor cut-to-surgery time | Each additional hour between graft cut and surgery is associated with a 0.36 μm decrease in baseline CCT. |

## Supplementary Online Table S4. Interpretation of Interaction Terms (Table 3)

| Interaction Term | Interpretation |
| --- | --- |
| Time ≤3 months | Represents the average rate of CCT reduction per day during the first 3 months after surgery. |
| Time >3 months | Represents the average rate of CCT change per day after 3 months. A near-zero or positive value suggests stabilization. |
| Surgery * Time ≤3 months | A positive interaction (β = 0.74 μm/day) indicates that CCT decreased more slowly in PKP vs DSAEK during the first 3 months. |
| Surgery * Time >3 months | No significant difference in the rate of CCT change between PKP and DSAEK beyond 3 months. |
| Donor age * Time ≤3 months | A positive interaction (β = 0.67 μm/day) means that grafts from older donors showed slower CCT reduction during the early post-op period. |
| Donor age * Time >3 months | No significant effect of donor age on the CCT change rate beyond 3 months. |
| ECD * Time ≤3 months | A negative interaction (β = -1.14 μm/day) indicates faster CCT reduction in grafts with higher donor ECD during the first 3 months. |
| ECD * Time >3 months | No significant difference in the rate of change after 3 months by donor ECD. |
| Graft thickness * Time ≤3 months | A negative interaction (β = -0.64 μm/day) suggests that thicker grafts had faster CCT reduction in the first 3 months. |
| Graft thickness * Time >3 months | No significant difference in the rate of change beyond 3 months by preoperative graft thickness. |
