# Supplementary material for: Impact of recipient and donor factors on corneal graft clearance: insights from serial anterior segment optical coherence tomography
Source: AJO Int. Author manuscript; Available in PMC 2026 Apr 7. (PMC13052495; doi:10.1016/j.ajoint.2025.100147)
Supplement: 4 [file NIHMS2107585-supplement-4.docx]

# Supplemental Appendix: Interpretation of Regression Model Outputs for Table 4

## Supplementary Online Table S5. Interpretation of Main Effects (Table 4)

| Covariate | Interpretation |
| --- | --- |
| Recipient age | Each additional year of recipient age is associated with a 0.71 μm decrease in baseline CGT. |
| Type of surgery | Not applicable (only DSAEK cases included). |
| Recipient pre-operative diagnosis (PACE vs Failed graft) | PACE was associated with 11.32 μm higher baseline CGT compared to failed graft, though not statistically significant. |
| Recipient pre-operative diagnosis (FECD vs Failed graft) | FECD was associated with 13.06 μm higher baseline CGT compared to failed graft, though not statistically significant. |
| Recipient pre-operative CCT | Each additional μm in pre-operative CCT is associated with a 0.004 μm increase in baseline CGT (not significant). |
| Donor age (≥56 vs <56) | Grafts from older donors were associated with 28.44 μm lower baseline CGT. |
| Donor diabetes status (Yes vs No) | Donor diabetes was associated with a non-significant 14.97 μm lower CGT. |
| Donor graft ECD (≥3021 vs <3021) | Grafts with higher donor ECD were associated with 51.80 μm higher baseline CGT. |
| Donor preoperative graft thickness (≥68.5 vs <68.5) | Thicker donor grafts were associated with 60.77 μm higher baseline CGT. |
| Donor death-to-preservation time | Each additional minute is associated with a 0.01 μm increase in CGT (not significant). |
| Donor death-to-surgery time | Each additional hour is associated with a 0.30 μm increase in CGT. |
| Donor cut-to-surgery time | Each additional hour is associated with a 0.08 μm decrease in CGT. |

## Supplementary Online Table S6. Interpretation of Interaction Terms (Table 4)

| Interaction Term | Interpretation |
| --- | --- |
| Time ≤3 months | Represents the average rate of CGT reduction per day during the first 3 months after surgery. |
| Time >3 months | Represents the rate of CGT change per day after 3 months. Near-zero values indicate stabilization. |
| Donor age * Time ≤3 months | Grafts from older donors showed a slower rate of CGT reduction during the first 3 months (β = 0.22 μm/day). |
| Donor age * Time >3 months | No significant difference in CGT change rate after 3 months by donor age. |
| ECD * Time ≤3 months | Grafts with higher donor ECD showed a faster rate of CGT thinning during the first 3 months (β = -0.42 μm/day). |
| ECD * Time >3 months | No significant difference in CGT change rate beyond 3 months by donor ECD. |
| Graft thickness * Time ≤3 months | Thicker grafts exhibited a faster early deturgescence rate (β = -0.42 μm/day). |
| Graft thickness * Time >3 months | No significant difference in CGT change rate beyond 3 months by graft thickness. |
